# Semantic-guided 3D Gaussian splatting for sparse-view reconstruction in industrial digital twins

**DOI:** 10.1186/s42492-026-00229-x

**Published:** 2026-08-17

**Authors:** Boyang Li, Tianhan Gao, Zuan Gu, Huimin Liu

**Affiliations:** Northeastern University, Shenyang, Liaoning 110167 China

**Keywords:** Industrial digital twin, Sparse-view reconstruction, 3D Gaussian splatting, Semantic guidance

## Abstract

Novel view synthesis, which is essential for three-dimensional (3D) reconstruction, traditionally requires dense image sets and pre-calibrated camera parameters. However, in industrial digital twin applications spatial constraints often limit data acquisition to sparse views that fail to provide the feature correspondences necessary for accurate modeling. To overcome this bottleneck, a semantic-guided 3D Gaussian splatting (3DGS) framework tailored to sparse-view industrial reconstruction was introduced. Rather than relying on precise initialization and dense inputs, the proposed method couples explicit 3D Gaussian representations with the dense and unconstrained stereo 3D reconstruction (DUSt3R) end-to-end pose estimation model. The severe occlusions, typical of factory environments, are handled by incorporating Segment Anything Model 2 (SAM2) to hierarchically decompose the scene, yielding a structured representation that improves optimization stability. A probability density field-driven algorithm is subsequently applied to extract lightweight meshes directly from the optimized Gaussian point clouds. Evaluations on the MipNeRF360 benchmark and a custom industrial dataset demonstrated substantial improvements in the structural similarity index measure. By enabling robust reconstruction from limited viewpoints, this pipeline offers a practical geometric foundation for automated inspection and remote equipment monitoring.

## Introduction

Modern industrial manufacturing is increasingly relying on digital twin systems to safely and efficiently manage complex, large-scale facilities [1]. Unlike static computer-aided design or building information modeling assets, digital twins enable bidirectional interaction and real-time state synchronization. This dynamic mapping is particularly crucial in hazardous environments, such as high-temperature or high-radiation zones, where manual inspection is costly and poses severe risks to human life. In these scenarios, high-fidelity three-dimensional (3D) reconstructions serve as the geometric foundation for unmanned monitoring, predictive maintenance, and remote execution [2].

However, constructing accurate 3D environments in real-world factories presents formidable challenges. Traditional pipelines, such as laser scanning and structure from motion (SfM) [3, 4], typically require dense image sets with over 60% overlap to establish reliable feature correspondences [5]. Factory floors are densely packed with equipment, which severely restricts camera placement and often limits data acquisition to a few sparse views. Furthermore, industrial equipment frequently exhibits intense metallic reflections and causes complex occlusions, which can degrade the completeness of traditional reconstruction methods by over 40% [5]. These factors invariably lead to topological holes, geometric distortions, and missing texture [6]. Compounding these issues is the quadratic computational complexity of SfM, which makes rapid deployment highly impractical.

Recent advancements in novel view synthesis offer potential solutions, yet limitations remain. Neural radiance fields (NeRFs) [7] utilize multilayer perceptrons (MLPs) for volume rendering; however, their slow training and inference speeds hinder practical industrial applications [8, 9]. Conversely, 3D Gaussian splatting (3DGS) [10] parameterizes scenes using explicit anisotropic 3D Gaussian primitives and differentiable rasterization to achieve real-time rendering [11]. Although 3DGS excels in dense inputs, its performance degrades precipitously under sparse-view conditions because of heavy dependency on accurate SfM initialization [12].

To bridge the gap between raw sparse data and interactive, lightweight digital representations [13], this paper proposes a semantic-guided sparse-view 3DGS framework tailored for industrial digital twins. This approach bypasses the initialization limitations of the traditional SfM by coupling explicit Gaussian representations with the end-to-end pose estimation model of dense and unconstrained stereo 3D reconstruction (DUSt3R) [14]. To handle metallic reflections and severe clutter, the vision foundation model SAM2 is integrated for semantic-guided hierarchical decoupling [15–17]. Finally, the optimized point clouds are processed into lightweight meshes, ensuring seamless execution on the Web or virtual reality platforms.

The core contributions of this work are summarized as follows:

- Sparse-view 3DGS optimization: A joint optimization pipeline is introduced to eliminate the dependency on dense inputs. By bridging explicit 3D Gaussian representations with globally aligned pose priors derived from DUSt3R, the framework achieves robust geometric initialization merely in seconds.
- Semantic-guidance hierarchical decoupling: SAM2 is leveraged to hierarchically decompose occluded industrial scenes. This isolates core equipment from environmental noise and lighting interference, guiding Gaussian convergence toward physically plausible solutions with improved boundary fidelity and structural coherence.
- Implicit-explicit hybrid modeling: A probability density field-driven mesh generation algorithm is designed to efficiently convert the optimized 3D Gaussian point clouds into lightweight, watertight mesh models.

**Fig. 1:**
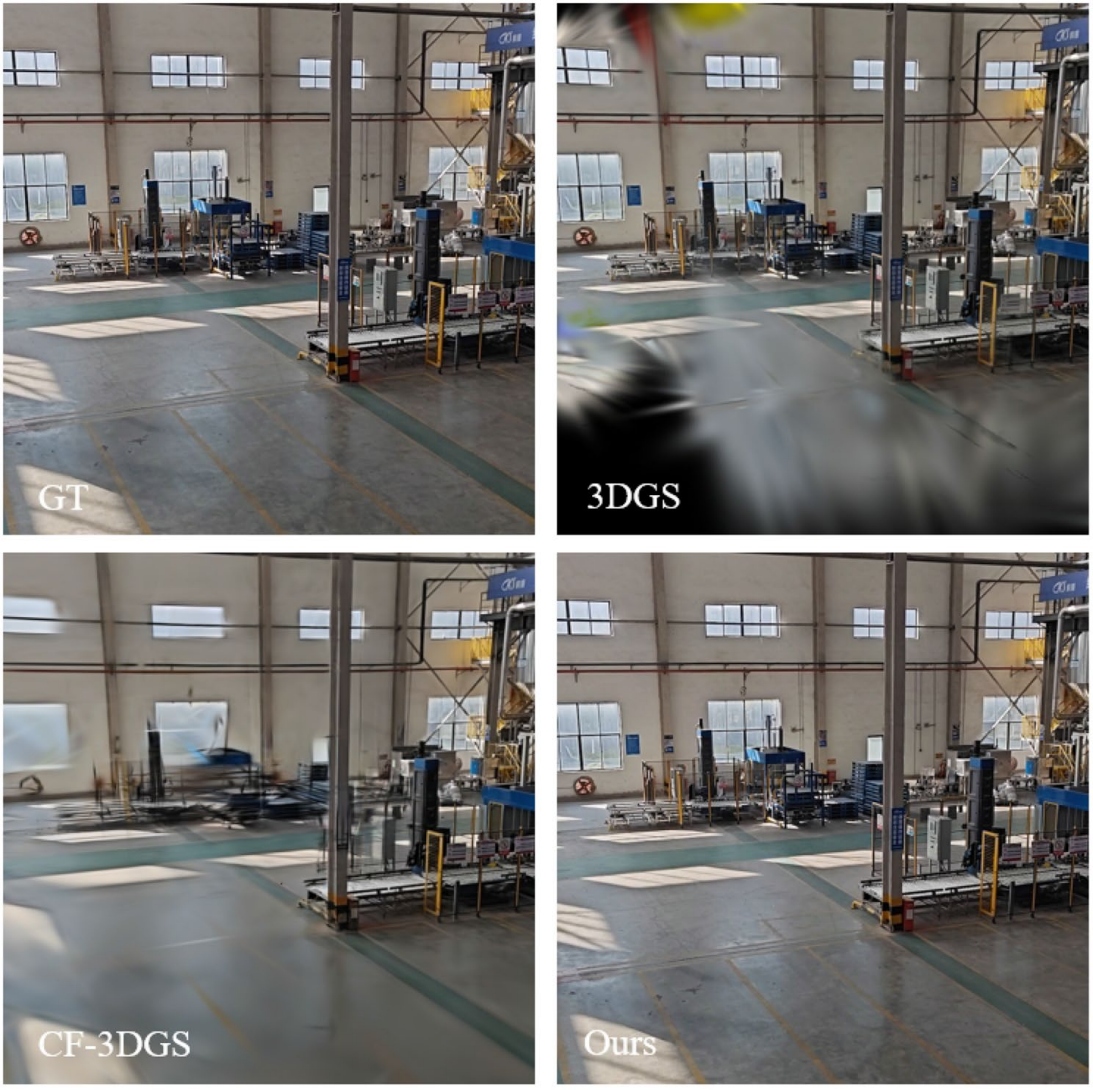
Comparative analysis under sparse 3-view input conditions. The proposed method achieves superior geometric consistency and detail fidelity compared to 3D Gaussian splatting (3DGS) and COLMAP-free (CF)-3DGS, with reconstructions significantly closer to ground truth references

The full pipeline completes full-scene reconstruction and view synthesis within 60 second. As illustrated in Fig. 1, this approach significantly improves the structural similarity index measure (SSIM) scores on both the MipNeRF360 benchmark and a custom industrial dataset, providing a highly reliable geometric basis for automated factory inspection and equipment health management.

### Sparse-view reconstruction

Sparse-view 3D reconstruction aims to synthesize photorealistic novel views from a limited set of perspectives, which is pivotal for cost-effective industrial digital twin development [7]. In NeRF [18], the computational inefficiency of scene representation through implicit MLPs hinders real-time industrial deployment. Although various NeRF-based methods have explored depth regularization [19–21] and vision-based priors [22–24] to mitigate sparse-view challenges, the high inference cost remains a significant bottleneck in practical applications [3].

Recently, 3DGS [25, 26], which replaces implicit MLPs with explicit anisotropic 3D Gaussian primitives [10], has emerged as a breakthrough. By combining parametric representation with differentiable rasterization, 3DGS achieves real-time rendering quality. Despite its efficiency, 3DGS suffers from severe over-smoothing and overfitting under sparse inputs [27]. This limitation primarily stems from the dependency on high-quality SfM initialization, which frequently fails in industrial scenarios due to insufficient feature correspondence. Recent attempts to mitigate these issues [12, 28] confirm that initialization quality remains the fundamental bottleneck in robust cross-scene generalization.

### Reconstruction model DUSt3R

Traditional 3DGS initialization heavily depends on COLMAP, a classic SfM pipeline that performs feature detection, matching, and bundle adjustment to estimate camera parameters and generate sparse point clouds. Under sparse-view conditions (* < *12 views), COLMAP exhibits dramatic degradation in point cloud accuracy because of insufficient feature correspondence [27], leading to flawed Gaussian initialization and subsequent reconstruction artifacts, such as geometric distortions and texture blurring. Similar robustness issues regarding feature selection and data association have also been extensively discussed in the literature on dynamic visual simultaneous localization and mapping [29].

The DUSt3R model [14] addresses this limitation through an end-to-end uncalibrated two-view reconstruction framework, capable of recovering camera poses and sparse point clouds within 2 second without pre-calibrated parameters. Compared to COLMAP, DUSt3R reduces peak graphics processing unit (GPU) memory consumption and achieves sublinear complexity growth with an increasing number of views [27], making it suitable for sparse-view industrial scenarios.

Notably, DUSt3R’s globally aligned geometric priors provide a critical solution to 3DGS’s initialization bottleneck. Recent works such as InstantSplat [27], which initialize Gaussian primitives on DUSt3R-derived point clouds, have demonstrated improved sparse-view performance; however, they lack semantic guidance for handling occluded industrial environments. The proposed framework addresses this gap.

### Semantic guided image segmentation

Semantic information plays a vital role in enhancing 3D reconstruction robustness, particularly for industrial scenes with complex occlusions and metallic reflections. In 3DGS, overlapping Gaussian primitives often cause blurred boundaries and detail loss, as the model struggles to distinguish object edges from noise under sparse inputs [12]. Early integration attempts using traditional segmentation methods failed to achieve satisfactory results because of limited generalization across industrial object categories [30]. In the medical imaging community, attention-augmented U-net variants such as deformable convolution and attention gate U-net have demonstrated that carefully designed dual-channel attention modules can significantly improve segmentation robustness under challenging noise and class imbalance conditions [31].

The emergence of transformer-based vision foundation models has revolutionized semantic segmentation. The segment anything model (SAM) [32] pioneered the “promptable segmentation” paradigm, enabling zero-shot generalization through large-scale training and interactive prompting. Its successor, SAM2 [33], further improved complex scene robustness via three key enhancements: (1) a dynamic weight fusion mechanism that aligns visual features with multimodal prompts (points, boxes, text) across transformer layers; (2) hierarchical prompt embedding that reduces computational complexity while preserving long-range dependencies; and (3) cross-view mask propagation enabled by epipolar geometry constraints. Compared to specialized models, such as Segment Everything Everywhere All at Once [34], which focus on universal segmentation and the model that segments everything in context, namely Segmentation Generative Pre-trained Transformer [35], SAM2 achieves state-of-the-art performance on Common Objects in Context [36], achieving a better balance between accuracy and efficiency.

Despite these advancements, the integration of SAM2-style semantic guidance with 3DGS remains underexplored in sparse-view industrial reconstruction. Existing works either rely on manual annotation or fail to leverage hierarchical semantic structures [37], which limits their ability to handle occluded equipment and cluttered factory environments, thereby motivating the semantic-guided decoupling strategy of this study.

Considering these, particularly the unreliable camera pose estimation and image registration from COLMAP under sparse-view conditions, this paper proposes a dual-model fusion framework for semantic-aware reconstruction. This framework integrates DUSt3R as a 3D prior model to provide globally aligned geometric initialization for 3D Gaussians and leverages the vision foundation model SAM2 for hierarchical decoupling of industrial scenes. This synergistic integration enables parallel optimization of 3D Gaussian attributes and camera parameters, effectively addressing the limitations of conventional approaches.

## Methods

**Fig. 2:**
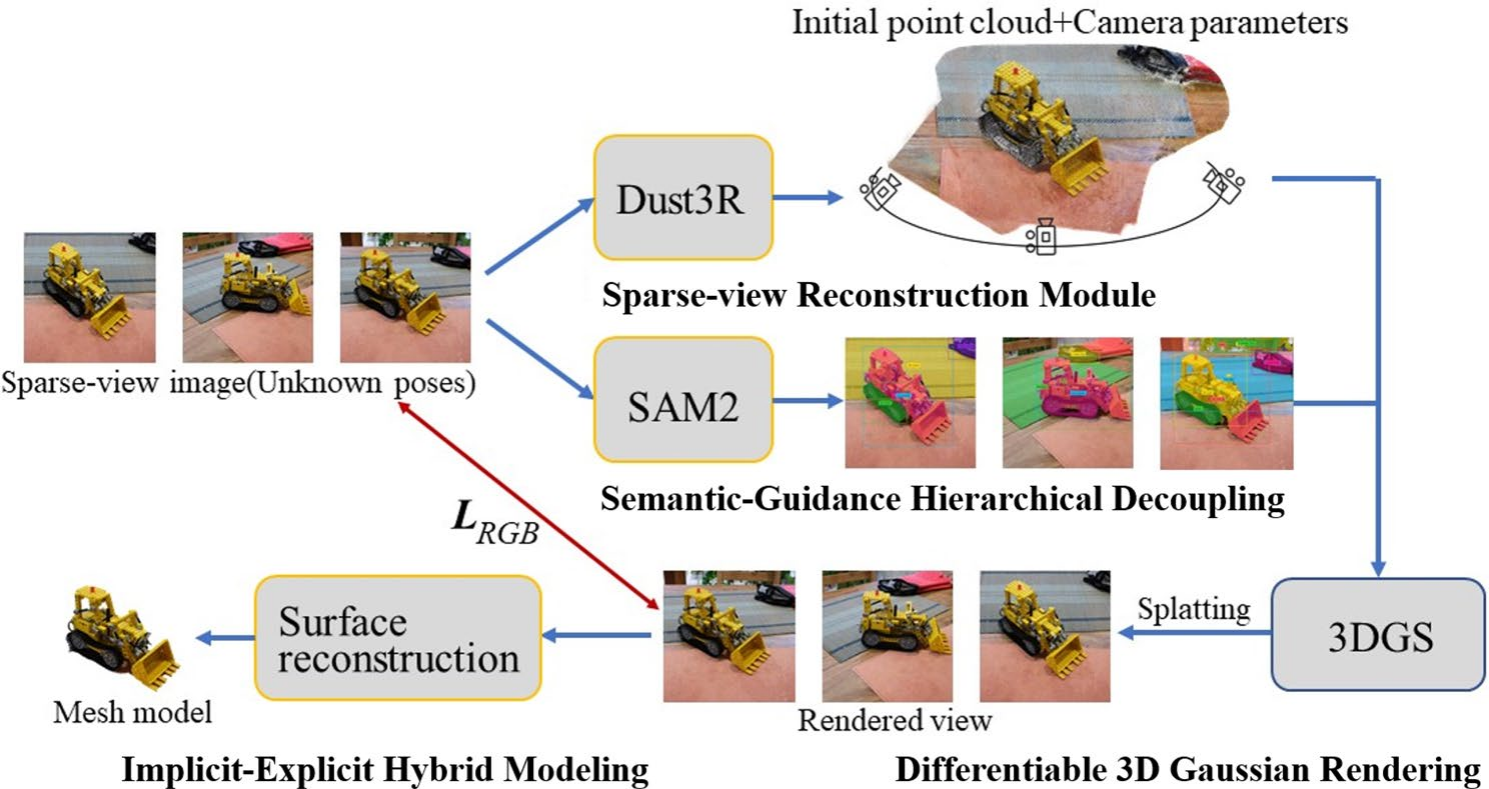
The proposed framework employs dense and unconstrained stereo 3D reconstruction to estimate camera parameters and initialize point clouds and 3D Gaussian primitives. Building upon this, SAM2 facilitates semantic comprehension and hierarchical geometric decoupling of input images. Ultimately, surface reconstruction coupled with mesh optimization is executed to enhance 3D reconstruction performance under sparse-view conditions. DUSt3R: Dense and unconstrained stereo 3D reconstruction; SAM: Segment anything model; 3DGS: 3D Gaussian splatting

This paper proposes an enhanced method based on the DUSt3R framework to address sparse 3D reconstruction requirements for complex power plant scenarios. The core innovation lies in integrating multimodal semantic constraints with robust geometric optimization. As illustrated in Fig. 2, the methodology comprises the following key components.

### Sparse-view 3DGS optimization

**Sparse-view reconstruction module.** The DUSt3R model generates initialization inputs for 3DGS. DUSt3R’s end-to-end reconstruction model takes two images as input and outputs per-pixel point maps and confidence maps. The ground-truth point maps, ${\widehat {\bf{P}}_{1,1}}$ and ${\widehat {\bf{P}}_{2,1}}$, obtained from the dataset, correspond to view pair (1,2), with the camera origin defined in View 1. The subscript (2,1) in ${\widehat {\bf{P}}_{2,1}}$ indicates that View 2’s coordinate system is anchored to View 1. The regression loss for DUSt3R training is defined as:

1 $$\mathcal{{\cal L}} = \left\| {\frac{1}{{{z_i}}} \cdot {{\bf{P}}_{v,1}} - \frac{1}{{{{\hat z}_i}}} \cdot {{\widehat {\bf{P}}}_{v,1}}} \right\|$$

where $v \in \{1,2\}$ denotes the view index; the ${\bf{P}}$ and $\widehat {\bf{P}}$ represent predicted and ground-truth values, respectively. To handle the scale ambiguity between predictions and the ground truth, DUSt3R normalizes the point maps using scaling factors ${z_i} = {\rm{norm}}({{\bf{P}}_{1,1}},{{\bf{P}}_{2,1}})$ and ${\hat z_i} = {\rm{norm}}({\widehat {\bf{P}}_{1,1}},{\widehat {\bf{P}}_{2,1}})$. Here, mark D refers to the distance from all valid points to the origin:

2 $$\mathcal{\rm{norm}}({{\bf{P}}_{1,1}},{{\bf{P}}_{2,1}}) = \frac{1}{{|{{\bf{D}}_1}| + |{{\bf{D}}_2}|}}\sum\limits_{v \in \{1,2\} } {\sum\limits_{i \in {{\bf{D}}_v}} {\left\| {{\bf{P}}_v^i} \right\|} } $$

For 3DGS initialization, both intrinsic and extrinsic camera parameters must be acquired. The Weiszfeld algorithm [38] computes focal lengths for each camera:

3 $$\mathcal{\textit{f} ^ *} = \arg {\min _f}\sum\limits_{i = 0}^W {\sum\limits_{j = 0}^H {{\omega ^{i,j}}} } \left\| {({i^\prime },{j^\prime }) - f\frac{{(P_0^{i,j},P_1^{i,j})}}{{P_2^{i,j}}}} \right\|$$

where ${i^\prime } = i - \frac{W}{2}$ and ${j^\prime } = j - \frac{H}{2}$ represent centered pixel indices, and *P* denotes the point-maps. The final camera focal length *\bar f* is obtained by averaging across all training views: $\bar f = mean({f^*})$.

To extend pairwise-aligned camera poses to global alignment, first, a fully connected graph ${{\cal P}}({{\cal V}},{{\cal E}})$ is constructed, with vertices ${{\cal V}}$ representing *N* input views and edges ${{\cal E}}$ that denote image pairs with shared visual content. For any image pair ${I_n}$,${I_m}$, the transformation matrices ${T_e}$, scaling factors ${\sigma _e}$, and globally aligned point maps *\tilde P* are optimized as follows:

4 $$\mathcal{\tilde P^ * } = \arg \mathop {\min }\limits_{\tilde P,T,\sigma } \mathop \sum \limits_{e \in {\cal E}} \mathop \sum \limits_{v \in e} \mathop \sum \limits_{i = 1}^{HW} \omega _{v,e}^i\tilde P_v^i - {\sigma _e}{T_e}P_{v,e}^i$$

where $\omega _{v,e}^i$ denotes the confidence weights, and $P_{v,e}^i$ represents the point-map projections for view *v* in edge *e*. To avoid trivial solutions (e.g., ${\sigma _e} = 0$), DUSt3R enforces the constraint $\mathop \prod \limits_e {\sigma _e} = 1$.

**Differentiable 3D Gaussian rendering.** The optimized point map ${\tilde P^ * }$ is converted into a 3D Gaussian representation:

5 $$\mathcal{G} = \{(\mu_i,\Sigma_i,w_i)| \mu_i\in\mathbb{R}^3, \Sigma_i\in\mathbb{S}_{++}^3, w_i\in\mathbb{R}^+\}$$

where$\Sigma_i=R_iS_iS_i^\top R_i^\top$ is parameterized by rotation matrices $R_i$ and anisotropic scaling $S_i$. The differentiable projection is implemented via the InstantSplat renderer [27]:

6 $$\frac{\partial \mathcal{L}_{\mathrm{render}}}{\partial \mathcal{G}} = \sum_{k=1}^N \|I_k - \sum_{i=1}^M w_i \mathcal{N}(\pi(\mathbf{P}_k\mu_i),\pi(\mathbf{P}_k\Sigma_i\mathbf{P}_k^\top))\|$$

This pipeline achieves parallelization through tiled compute unified device architecture kernels, attaining real-time rendering at 30frames per second on NVIDIA RTX 4090 platforms.

### Semantic-guidance hierarchical decoupling

This module leverages the vision foundation model SAM2 to achieve hierarchical decoupling of industrial scenes, providing structured representations for core reconstruction tasks. Given a sparse image sequence $\mathcal{I}=\{I_t\}_{t=1}^T \subset \mathbb{R}^{H\times W \times 3}$, the system first employs SAM2’s prompt encoder to map user-provided spatial coordinates $\mathcal{P}_t=\{(x_i,y_i)\}_{i=1}^k$ into semantic query vectors:

7 $$\mathbf{q}_t = \sum_{i=1}^k \gamma(\mathrm{PE}(x_i,y_i)) \odot \mathcal{F}_t(x_i,y_i)$$

where $\mathrm{PE}(\cdot)$ denotes Fourier positional encoding; $\gamma: \mathbb{R}^d \to \mathbb{R}^d$ is a lightweight adapter; and $\mathcal{F}_t \in \mathbb{R}^{\frac{H}{16}\times\frac{W}{16}\times256}$ represents the multiscale features generated by the SAM2 encoder. This design enables the system to adapt to industrial scene characteristics through parameter-efficient fine-tuning of lightweight adapters, while largely preserving the pretrained model’s zero-shot capabilities.

**Fig. 3:**
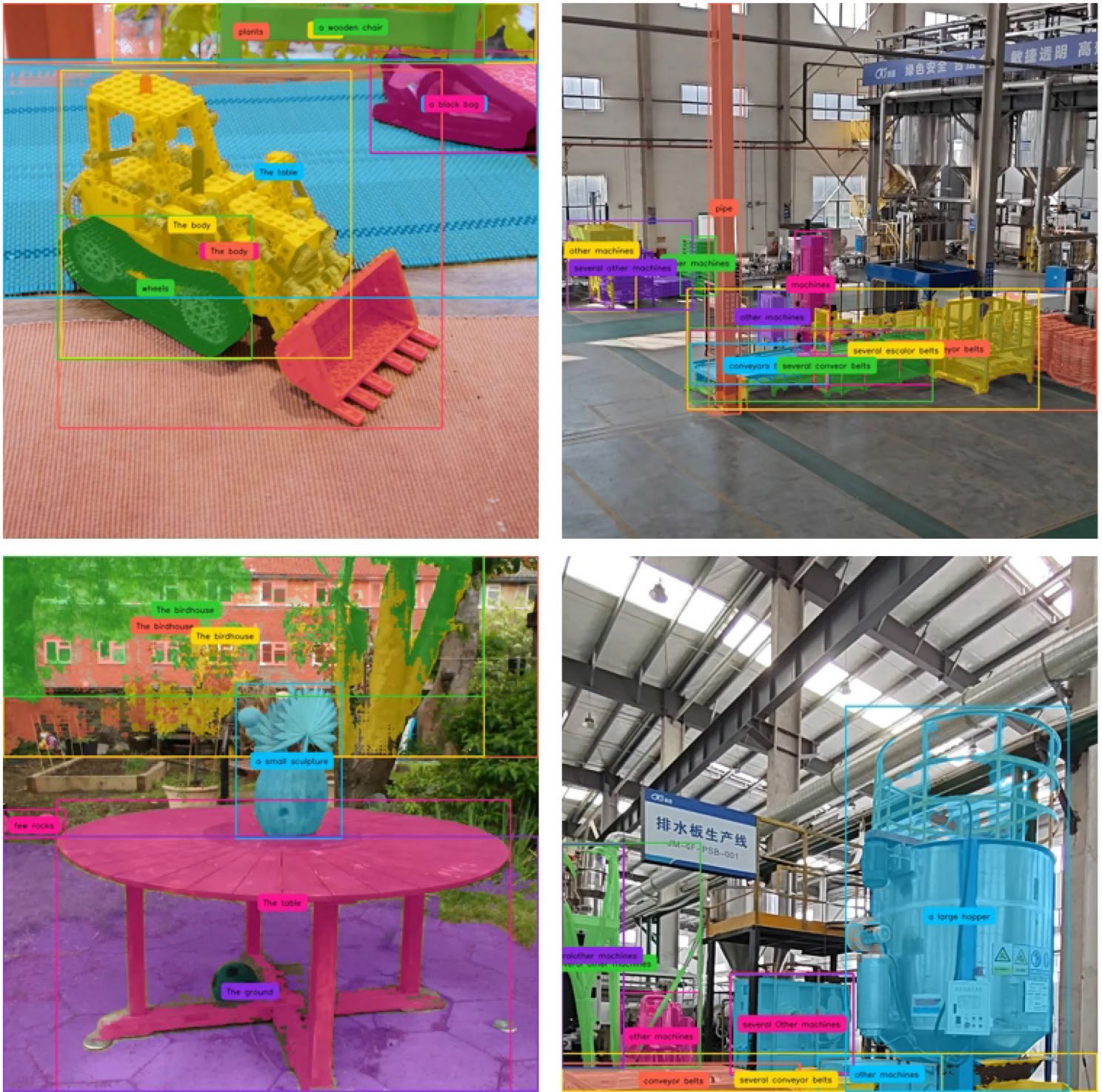
Semantic-guidance hierarchical decoupling successfully guides the hierarchical decomposition of scenes

The mask decoder generates foreground segmentation results through cross-layer attention mechanisms based on the query vector $\mathbf{q}_t$:

8 $$\mathbf{M}_t^{\mathrm{fg}} = \sigma\left(\sum_{l=1}^L \mathrm{Softmax}\left(\frac{\mathbf{q}_t W_Q (\mathcal{F}_t^{(l)} W_K)^\top}{\sqrt{d}}\right) \mathcal{F}_t^{(l)} W_V\right)$$

where $W_Q, W_K, W_V \in \mathbb{R}^{d \times d}$ are learnable projection matrices, and *L* = 8 indicates the number of attention layers. To address the occlusion challenges in sparse-view scenarios, epipolar geometry constraints are introduced to establish a cross-view mask propagation model. For adjacent view pairs $(I_t, I_{t+1})$, temporal consistency of masks is achieved through optical flow estimation of deformation fields $\mathbf{F}_{t \to t+1} \in \mathbb{R}^{H\times W\times 2}$:

9 $$\mathbf{M}_{t+1}^{\mathrm{fg}} = \mathcal{W}(\mathbf{M}_t^{\mathrm{fg}}, \mathbf{F}_{t \to t+1}) \odot \mathbf{V}_{t \to t+1}^{\mathrm{vis}}$$

where $\mathbf{V}_{t \to t+1}^{\mathrm{vis}} \in \{0,1\}^{H\times W}$ denotes the depth-based visibility mask, and $\mathcal{W}(\cdot)$ represents differentiable bilinear sampling. This process fuses multiview predictions into a unified representation $\hat{\mathbf{M}}_t^{\mathrm{fg}} = \bigcup_{\tau \in \mathcal{N}(t)} \pi_{t \to \tau}(\mathbf{M}_\tau^{\mathrm{fg}}),$ where $\pi_{t \to \tau}$ indicates camera pose-based projective transformations. The hierarchical reconstruction stage processes the decoupled foreground $\mathcal{I}^{\mathrm{fg}} = \{\mathbf{M}_t^{\mathrm{fg}} \odot I_t\}$ and background $\mathcal{I}^{\mathrm{bg}} = \{(1-\mathbf{M}_t^{\mathrm{fg}}) \odot I_t\}$, which are then fed into the sparse-view 3DGS optimization module for Gaussian point cloud generation. The image decoupling effect is shown in Fig. 3.

### Implicit-explicit hybrid modeling

**Surface reconstruction framework based on probability density fields.** Surface reconstruction from probability density fields employs a variational implicit surface reconstruction framework to convert the discrete and potentially noisy Gaussian point cloud representation into a continuous, smooth, and watertight mesh. This approach defines the target surface as the zero-level set of a scalar-valued implicit function $f: \mathbb{R}^3 \to \mathbb{R}$. The function *f* is constructed based on the probability density field induced by the optimized 3D Gaussians:

10 $$ f(x) = \sum_{i=1}^{N} \exp \left(-\frac{1}{2}(x - p_i)^T \Sigma_i^{-1}(x - p_i)\right)$$

where $\{p_i, \Sigma_i\}_{i=1}^N$ are the centers and covariances of the Gaussians, respectively. The surface is then extracted by finding the isosurface *f(x) = t* for a given density threshold *t*.

To determine the optimal implicit function *f*, an energy minimization problem was formulated. This problem is inherently ill-posed, as infinitely many functions can satisfy the data constraints. Therefore, regularization is essential to ensure a unique and well-behaved solution, particularly in regions with sparse or noisy data. Therefore, the optimization objective combined a data fidelity term with a hybrid regularization term:

11 $$ \min_{f} \sum_{x \in S} |f(x) - t|^2 + \mathcal{L}_{\mathrm{reg}}$$

where *S* is a set of points sampled from the space to enforce the constraint, and $\mathcal{L}_{\mathrm{reg}}$ is the carefully designed regularizer.

The regularization term, $$\mathcal{L}_{\mathrm{reg}} = \lambda_1 ||\nabla f||_2 $$$$+ \lambda_2 ||\nabla^2 f||_F$$, consists of two complementary components that work in synergy. The first term, $$\lambda_1 ||\nabla f||_2$$, is a first-order regularizer based on the Dirichlet energy. In the context of learning implicit representations, this term acts as a soft Eikonal constraint, encouraging the gradient of the implicit field to have a uniform magnitude near the surface, which is a fundamental property of signed distance functions.

However, first-order priors alone are known to be insufficient for ensuring higher-order smoothness and can produce undesirable artifacts such as sharp creases. Therefore, a second-order regularization term, $\lambda_2 ||\nabla^2 f||_F$, that penalizes the Frobenius norm of the function’s Hessian matrix was introduced. This term was inspired by thin-plate spline models and enforces $C^2$ smoothness, ensuring a smoothly varying normal field across the surface. Crucially, these two terms are not redundant. The first-order term primarily governs the behavior of the implicit function on and near the data points, whereas the second-order term dominates away from the data, forcing the gradient field to remain constant, thereby preventing the formation of spurious surface sheets in empty space. This hybrid approach is essential for robustly handling the noisy and incomplete point clouds generated by 3DGS and is consistent with state-of-the-art practices in neural surface reconstruction that combine Eikonal and smoothness losses for stability and fidelity.

**Fig. 4:**
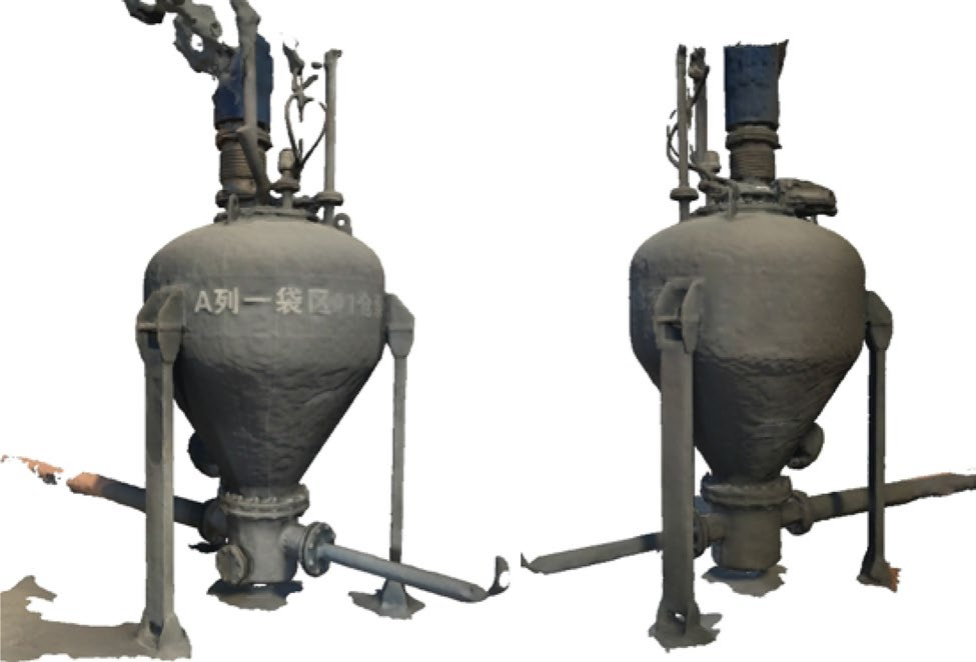
Surface reconstruction results

The final optimized implicit function *f* provides a continuous and smooth representation of the scene geometry. Multi-resolution hash encoding was employed to accelerate spatial discretization and extract the final mesh $M_0(V_0, F_0)$, using a differentiable marching cubes algorithm. The surface reconstruction results that are based on probability density fields are demonstrated in Fig. 4.

### Experimental setup

**Datasets.** The proposed method was evaluated on the MipNeRF360 [39] and custom industrial scene datasets. The MipNeRF360 dataset provides complex large-scale environments with wide viewing angles. The custom industrial dataset comprises factory inspection scenarios captured using consumer-grade smartphone cameras (1024 *×* 1024 resolution), simulating real-world industrial inspection workflows. This dataset contains intricate industrial equipment and occluded scenes, with all the images acquired without pre-calibrated camera intrinsics or extrinsics.

**Metrics.** The 3D reconstruction quality for both datasets was assessed by employing two standard metrics: SSIM [40] to quantify the structural similarity between rendered and ground-truth images and learned perceptual image patch similarity (LPIPS) [41] to measure the perceptual differences through deep feature comparisons.

**Implementation details.** The implementation used PyTorch with training/inference on an NVIDIA RTX 4090 GPU. Other hardware configurations included an Intel i9-13900 CPU and 64 GB of RAM. The model was subjected to adaptive moment estimation (Adam) for 1000 iterations. The pre-trained DUSt3R model was used, initializing it at a 1024 *×* 1024 resolution, with multiview geometry constraints implemented through differentiable Gauss-Newton optimization.

**Baselines.** Comparative experiments included: 3DGS [10]: Explicit 3D Gaussian splatting requiring COLMAP-derived camera parameters. CF-3DGS [37]: Single-view initialized Gaussian reconstruction that incrementally incorporates training views without COLMAP dependencies, aligning with the camera-agnostic design of the proposed approach.

## Results and discussion

### Experimental results

**Fig. 5:**
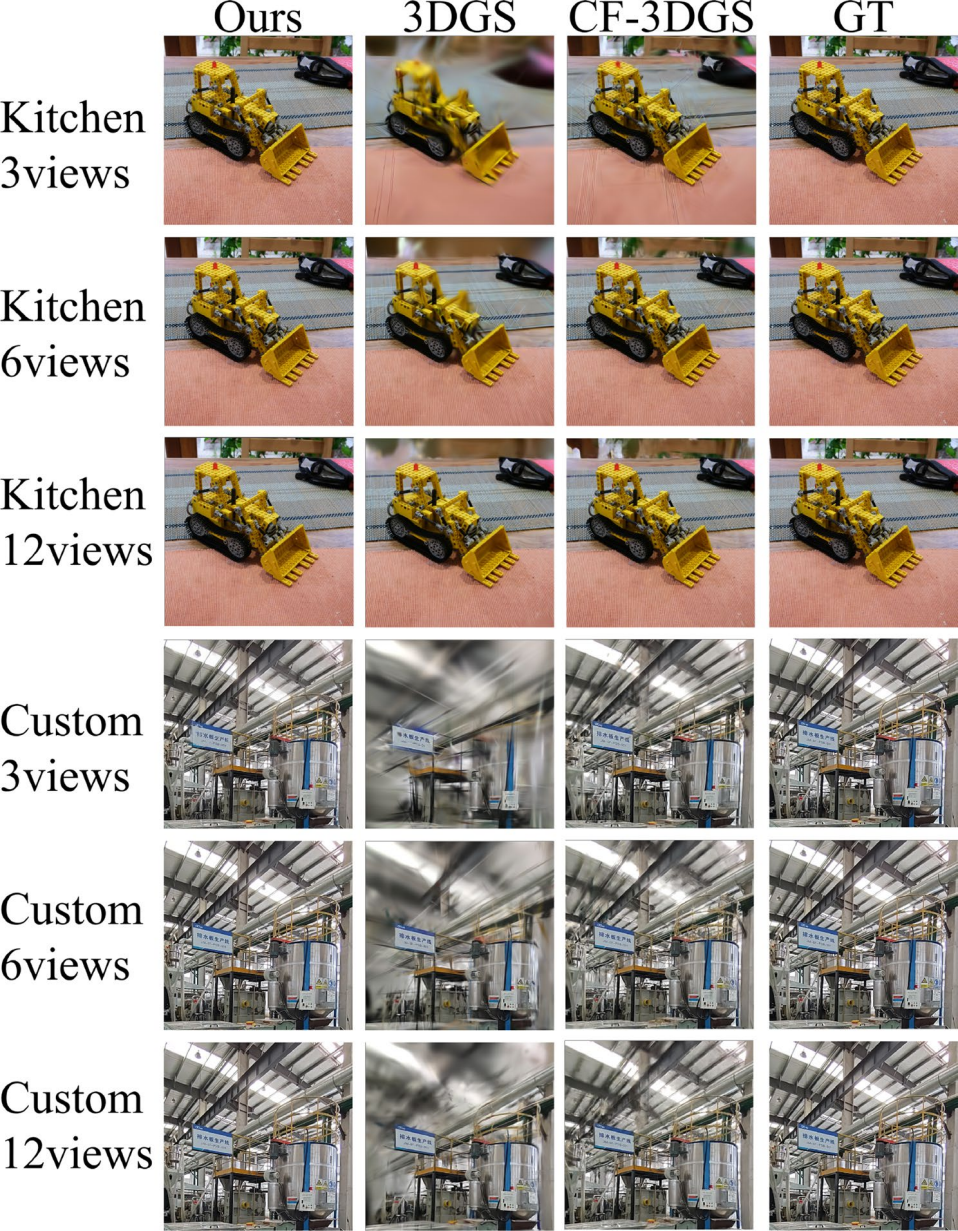
Qualitative results on the MipNeRF360 and custom industrial datasets demonstrate that the proposed method effectively preserves scene details while mitigating the artifacts caused by sparse input views in 3D Gaussian splatting. Compared with COLMAP-free 3D Gaussian splatting, the proposed method generates sharper reconstructions by introducing semantic priors. 3DGS: 3D Gaussian splatting; CF-3DGS: COLMAP-free 3D Gaussian splatting; GT: Ground-truth

**Fig. 6:**
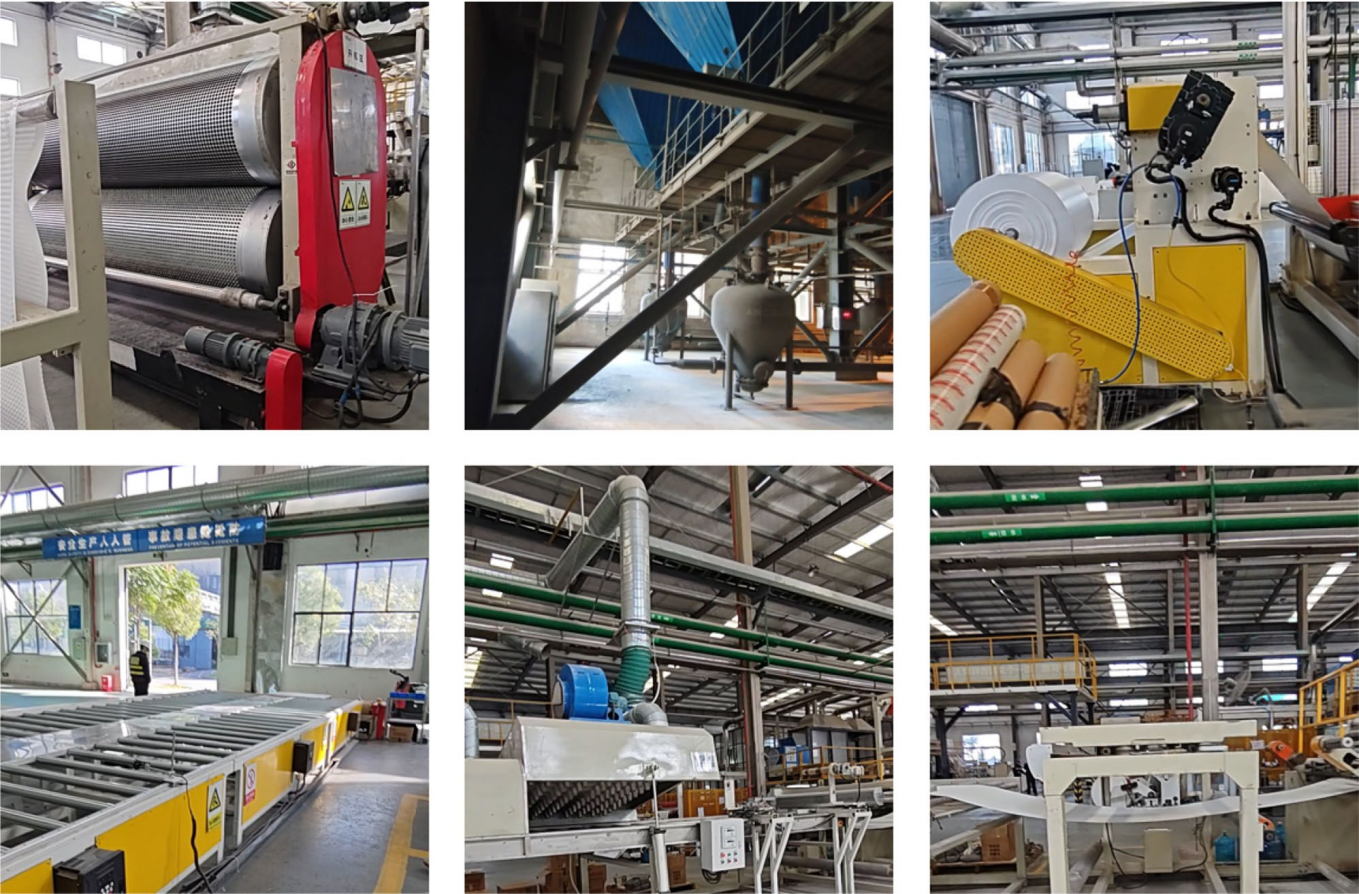
Under the 12-view configuration of the custom industrial dataset, the proposed method produces no obvious blurring or artifacts across diverse real-world industrial scenarios in experimental results, thereby meeting the requirements of digital twin applications

**Quantitative and qualitative results.** As demonstrated in Tables 1 and 2, the proposed method consistently outperformed alternative approaches under 3/6/12-view configurations. The qualitative analysis in Fig. 5 reveals significant disparities in visual reconstruction quality across the methods. Compared to ground-truth imagery, traditional 3DGS struggles with detail recovery and geometric consistency, particularly under sparse-view conditions, exhibiting blurring artifacts and structural distortions. CF-3DGS similarly underperforms in sparse-view scenarios. This limitation stems from error-prone pose predictions caused by the complex optimization process under limited viewing angles. Figure 6 illustrates the application of the proposed method across various real-world industrial scenarios.

**Table 1:** Quantitative evaluation under the 3/6/12 views of the MipNerf360 dataset; the proposed method is superior to other methods

Scene	Ours		3DGS		CF-3DGS	
	**SSIM***↑*	**LPIPS***↓*	**SSIM***↑*	**LPIPS***↓*	**SSIM***↑*	**LPIPS***↓*
Kitchen 3views	0.839	0.055	0.589	0.442	0.758	0.241
Kitchen 6views	0.852	0.051	0.813	0.174	0.836	0.054
Kitchen 12views	0.884	0.048	0.858	0.101	0.879	0.049
Garden 3views	0.791	0.067	0.341	0.354	0.615	0.157
Garden 6views	0.826	0.061	0.661	0.276	0.812	0.073
Garden 12views	0.838	0.052	0.713	0.122	0.835	0.067

SSIM: Structural similarity index measure; LPIPS: Learned perceptual image patch similarity; 3DGS: 3D Gaussian splatting; CF-3DGS: COLMAP-free 3D Gaussian splatting

**Table 2:** Quantitative evaluation under the 3/6/12 views of the custom dataset; the proposed method is superior to other methods

Scene	Ours		3DGS		CF-3DGS	
	**SSIM***↑*	**LPIPS***↓*	**SSIM***↑*	**LPIPS***↓*	**SSIM***↑*	**LPIPS***↓*
Tank 3views	0.834	0.049	0.252	0.557	0.333	0.185
Tank 6views	0.853	0.036	0.487	0.400	0.573	0.142
Tank 12views	0.894	0.037	0.417	0.376	0.761	0.137
Factory 3views	0.849	0.056	0.337	0.502	0.412	0.177
Factory 6views	0.877	0.036	0.459	0.437	0.641	0.129
Factory 12views	0.914	0.031	0.411	0.314	0.732	0.118

SSIM: Structural similarity index measure; LPIPS: Learned perceptual image patch similarity; 3DGS: 3D Gaussian splatting; CF-3DGS: COLMAP-free 3D Gaussian splatting

The proposed framework leverages the DUSt3R pose estimation model to acquire initial scene geometry from sparse views, whereas the SAM2 model provides structured representations for core reconstruction tasks. This synergistic integration delivers a streamlined and effective solution. Across all tested scenarios, the proposed approach achieves superior visual fidelity, with sharper details and enhanced geometric alignment.

### Ablation studies

This subsection systematically validates the efficacy of each module through controlled ablation experiments, strictly adhering to the principle of independent variables. All experiments were conducted under identical hardware configurations (NVIDIA RTX 4090 GPU) on the custom industrial scene dataset, with the results averaged over five trials to ensure statistical reliability.

#### Sparse-view reconstruction module analysis

To comprehensively evaluate the multiview reconstruction module, rigorous comparative experiments were conducted on the industrial dataset. For the experimental group, the proposed DUSt3R-based framework was utilized, whereas for the control group, the traditional SfM pipeline COLMAP was employed. Reconstruction quality, computational efficiency, and resource consumption were systematically compared under 3/6/12-view configurations on unified hardware.

As shown in Table 3, the proposed method demonstrates significant advantages across all metrics. With 12 input views, DUSt3R achieves a 46.0% SSIM improvement (0.883 vs 0.605) and 81.6% LPIPS reduction (0.042 vs 0.228) over COLMAP, proving that transformer-based feature aggregation better preserves scene details and geometric consistency. Computational efficiency exhibits sublinear growth with increasing views when scaling from three to 12 views, with DUSt3R’s processing time increasing marginally compared to COLMAP’s quadratic complexity. This scalability stems from the hierarchical attention mechanism: local window attention extracts fine-grained features, whereas global cross-view attention establishes robust correspondences, avoiding the combinatorial explosion of feature matching in traditional SfM.

**Table 3:** Sparse-view reconstruction module ablation studies

Method	View	SSIM*↑*	LPIPS*↓*	Time (minute)*↓*	Memory (GB)*↓*
COLMAP	3	0.380	0.460	24.2	4.2
Ours	3	**0.828**	**0.051**	**2.8**	**3.6**
COLMAP	6	0.600	0.320	34.1	6.8
Ours	6	**0.853**	**0.046**	**5.1**	**4.9**
COLMAP	12	0.605	0.228	67.6	11.2
Ours	12	**0.883**	**0.042**	**8.4**	**6.4**

Bold entries denote the results of the proposed method (ours), which achieves the best SSIM, LPIPS, runtime, and memoryusage under each view configuration

Regarding memory management, DUSt3R reduces peak GPU memory consumption by 42.9% (6.4 GB vs 11.2 GB) compared with COLMAP under the same view settings. The results confirm the superiority of the proposed method in factory scene reconstruction, attributable to the end-to-end differentiable architecture eliminating iterative SfM optimization, explicit geometric constraint modeling via cross-view attention, and redundant computation minimization.

**Semantic-guidance hierarchical decoupling.** To validate the core value of the SAM2 module, rigorous two-group controlled experiments were conducted on the custom dataset: (1) full method with SAM2 integration and (2) manual semantic segmentation baseline. Systematic evaluations were performed across three dimensions: semantic-geometric decoupling accuracy, interactive efficiency, and geometric preservation, using mean intersection over union as the primary segmentation metric.

As shown in Table 4, segmentation quality is comparable between manual annotation and SAM2-assisted approaches. The proposed method achieves substantial efficiency gains: manual annotation requires approximately *8.9×* longer interaction time than SAM2-assisted labeling (18.7 vs 2.1 minute) due to polygon drawing overhead. Notably, the baseline method generates fragmented regions through post-annotation geometric clustering, whereas SAM2’s visual prompt mechanism produces more coherent instance-level masks, reducing the surface deformation error from 0.32 to 0.15 (Table 4).

**Table 4:** Semantic-guidance module ablation studies

Method	mIoU (%)*↑*	Edit time (minute)*↓*	Deformation error*↓*
SAM2	**62.4**	**2.1**	**0.15**
Manual mark	64.7	18.7	0.32

Bold entries denote the results of the SAM2-integrated approach, which achieves superior interaction efficiency and geometricpreservation compared with the manual annotation baseline. mIoU: Mean intersection over union

The results demonstrate that the proposed framework effectively unifies semantic understanding with geometric decoupling through SAM2, thereby resolving the inherent semantic-geometric discrepancy in traditional methods. This integration provides structured representations for reconstruction tasks while enhancing robustness in occluded scenarios.

#### Parameter sensitivity analysis

To evaluate the robustness of the sparse-view initialization strategy, the impact of the confidence threshold (*τ*) used during the point cloud generation phase was analyzed. This parameter filters the raw predictions from the DUSt3R model, determining the density and reliability of the initial 3D Gaussians. All experiments were conducted on the “factory 6views” scene from the custom dataset.

The model outputs a confidence map *C ∈ [0, 1]* for each pixel, indicating the reliability of the estimated 3D coordinates. The initial point cloud $\mathcal{P}_{init} = \{p_i \mid C_i > \tau\}$ is filtered by varying *τ* across $\{0.2, 0.4, 0.5, 0.6, 0.8\}$. This threshold directly governs the trade-off between the completeness of scene coverage and precision of geometric initialization.

**Fig. 7:**
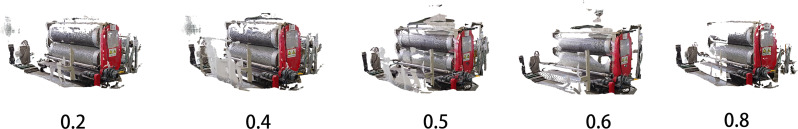
Qualitative impact of the confidence threshold (*τ*) on initialization quality. Lower thresholds (*τ=0.2*) introduce excessive noise and floating artifacts, whereas higher thresholds (*τ=0.8*) lead to significant structural loss and missing geometries. The selected threshold of *τ=0.5* achieves the optimal balance, preserving structural completeness without including unreliable noise

The impact of *τ* was assessed considering two aspects: the quantity of initial points (initialization density) and final reconstruction quality (SSIM). As illustrated in Fig. 7, the results in Table 5 indicate the following:

**Table 5:** Quantitative and qualitative sensitivity analysis of confidence threshold *τ* on the “factory 6views” scene

Threshold (*τ*)	Initial point	**SSIM***↑*	Qualitative observation
0.2	Approximately 125,000	0.858	Noisy: Floaters in air; blurry edges.
0.4	Approximately 85,000	0.871	Good: Dense coverage, minimal noise.
**0.5 (Ours)**	Approximately 68,000	**0.877**	**Best: Clean geometry and sharp details.**
0.6	Approximately 52,000	0.873	Good: Slightly sparse but clean.
0.8	Approximately 35,000	0.849	Incomplete: Missing parts in textureless areas.

Note: Bold entries denote the adopted confidence threshold (*τ* = 0:5) and the corresponding peak initialization density, SSIM, andqualitative performance

Setting a lenient threshold (*τ < 0.3*) preserves more points (approximately 120,000 points at *τ=0.2*) but introduces significant outliers and floating noise from low-confidence regions (e.g., sky or textureless walls). This noisy initialization forces the 3DGS optimization to expend capacity on correcting geometry rather than refining details, leading to suboptimal SSIM scores.

An overly strict threshold (*τ > 0.7*) discards valid geometric information, resulting in an overly sparse initialization (approximately 35,000 points at *τ=0.8*). Although the remaining points are highly accurate, the lack of coverage in valid areas causes ‘holes’ that the Gaussian splitting process fails to fully recover within the limited iteration budget, degrading visual fidelity.

The method achieves peak performance at *τ = 0.5*. At this level, the initialization provides sufficient scene coverage while effectively filtering out unreliable predictions, thereby serving as an ideal starting point for high-fidelity Gaussian splatting.

## Conclusions

A novel sparse-view 3D scene reconstruction framework is proposed to effectively address the challenges associated with data sparsity in 3D Gaussian splatting-based methods. By introducing DUSt3R-based initialization and semantically guided hierarchical decoupling, the proposed approach significantly enhances reconstruction accuracy and robustness in scenarios with limited input views. This methodology successfully strengthens geometric consistency, mitigates noise artifacts, and preserves fine-grained details. Experimental results across multiple benchmark datasets demonstrate consistent gains over 3DGS, CF-3DGS, and COLMAP-based baselines in SSIM and LPIPS (Tables 1, 2, and 3). The framework exhibits robust performance in various challenging environments, particularly in complex industrial scenarios characterized by metallic reflections and occlusions.

Future research will focus on temporally consistent dynamic scene reconstruction, leveraging inter-frame Gaussian parameter prediction, to extend sparse reconstruction to dynamic industrial processes, thereby enabling rapid reconstruction of time-evolving scenes. Dynamic 3D scene reconstruction will be further applied to digital twin systems for real-time equipment health monitoring and predictive maintenance.

## Data Availability

The datasets employed in this study are available from the corresponding authors upon reasonable request. In compliance with licensing requirements, the MipNeRF360 dataset is publicly accessible. Our custom industrial scene dataset was collected through on-site photography in industrial facilities.

## Abbreviations

3D: Three-dimensional
3DGS: 3D gaussian splatting
CF-3DGS: COLMAP-free 3D Gaussian splatting
DUSt3R: Dense and unconstrained stereo 3D reconstruction
GPU: Graphics processing unit
LPIPS: Learned perceptual image patch similarity
mIoU: Mean intersection over union
MLP: Multilayer perceptron
NeRF: Neural radiance field
SAM: Segment anything model
SfM: Structure from motion
SSIM: Structural similarity index measure
